# A new species of *Octaspidiotus* (Hemiptera, Diaspididae) from China

**DOI:** 10.3897/zookeys.605.8944

**Published:** 2016-07-14

**Authors:** Jiufeng Wei, Qing Zhao

**Affiliations:** 1 College of Agriculture, Shanxi Agricultural University, Taigu, Shanxi, China

**Keywords:** Taxonomy, Sternorrhyncha, armored scale insect, China

## Abstract

Adult females of a new species of armored scale insect, *Octaspidiotus shanghaiensis* **sp. n.** are described and illustrated from specimens collected in China. A key is provided for the all described species of *Octaspidiotus*.

## Introduction

Scale insects (Hemiptera: Coccoidea) are sap-sucking parasites which are small (generally less than 5 mm) and cryptic in their habitats (Gullan 1997), with at least 30 families and approximately 8000 species (García et al. 2016). Containing more than 2500 described species, Diaspididae is the largest species-rich family in the Coccoidea (García et al. 2016). Adult diaspidid females are sessile and permanently reside on their host plants (Gullan 1997). Adult females have the complete loss of the legs, the reduction of the antennae to a single segment and the modification of the abdomen into a specialized pygidium for forming the test, and these characteristics are the primary recognition features for these insects (Andersen 2010; Balachowsky 1948). Armored scale insects are important agricultural pests and have colonized a diverse set of plant species. They are distributed on every continent except Antarctica (Andersen 2010).

Although the family classification is controversial, the Aspidiotinae and the Diaspidinae are the two major subfamilies. The genus *Octaspidiotus* was established as a member of the former subfamily by MacGillivray (1921), with *Aspidiotus subrubescens* Maskell as its type species. However, two species that he transferred from *Aspidiotus* are not now included in this genus. Since then, many additional species were described and added to *Octaspidiotus* by other authors (Borchsenius 1966; Tang 1984; Tang and Chu 1983; Takagi 1984).

Takagi (1984) showed that *Octaspidiotus corticoides* (Green) was not a member of *Octaspidiotus* because the distinguishing characteristics were invalid. Currently, this genus is comprised of 14 valid species, eight of which are known to occur in China (García et al. 2015; Tang 1984; Takagi 1984). There are only two species recorded from Oceania, the other 12 species being distributed throughout East Asia.

Recently, one new species of *Octaspidiotus* was discovered from China. It was described and illustrated in this paper, bringing the number of recognized species in the genus to 15, of which nine species are recorded from China. A key to all known species of *Octaspidiotus* is provided.

## Materials and methods

In this paper, the terminology described by Henderson (2011) has been used. This publication also includes illustrations for most of the species treated herein. All measurements are presented in micrometers (μm). Measurements were made using NIT-Elements D tools.

The abbreviations L_1_, L_2_ and L_3_ are short for the median, second, and third pygidial lobes, respectively.

All specimens have been deposited in the Entomological Museum, Northwest A & F University, Yangling, Shaanxi, China (NWAFU).

## Taxonomy

### Genus Octaspidiotus

Taxon classificationAnimaliaHemipteraDiaspididae

MacGillivray, 1921

Metaspidiotus Takagi, 1957: 35. Junior synonym.

#### Type species.

*Aspidiotus subrubescens* Maskel, 1892.

#### Generic diagnosis.

**Adult female.** Body is oval to rounded; derm membranous except pygidium. **Cephalothorax.** Antennae with 1 seta. No trilocular pores associated to the spiracles. **Pygidium.** With 3-4 pairs of lobes, never bilobed. Median lobes (L_1_) well-developed, with notches on both margins or only present on the outer margin. Second lobes (L_2_) smaller than L_1_, with notches on both laterals or only present on the outer lateral. Third lobes (L_3_) similar to L_2_. Fourth lobes (L_4_) small and pointed apically, only present in *Octaspidiotus subrubescens*. Marginal setae occurring on dorsal bases of L_2_ and L_3_, lanceolate, broadened and flattened. Plates are well-developed, fimbriate on the outer margin in most species, occurring laterally and even extended to the abdominal segment IV. Paraphyses absent on pygidial margin. **Ducts.** Dorsum has one-barred type macroducts, that are aligned in some species. Ventral microducts are scattered. **Anal opening** is toward the apex of the pygidium, more or less elongate. Vulvar opening situated anterior to anal opening. **Perivulvar pores** are quinquelocular, present or absent, if present, in four groups.

#### Remarks.

This genus is very close to *Aspidiotus* Bouché, 1833 and *Oceanaspidiotus* Takagi, 1984 in terms of pygidial lobes and pygidium, but can be distinguished by the form of the dorsal marginal setae occurring on L1 and L2 which are lanceolate, broadened and flattened, while these setae in the other two genera are simply thickened.

### Octaspidiotus shanghaiensis sp. n.

Taxon classificationAnimaliaHemipteraDiaspididae

http://zoobank.org/07E3AD76-AF7A-4130-92CC-0C7895FF0A0F

Figures 1–7

#### Material examined.

**Holotype**: 1 adult female: CHINA: Changfeng Park, Shanghai City, 11. IV. 2015, Hongliang Li (NWAFU).

**Paratypes**. 3 adult females: same data as the holotype (NWAFU).

#### Diagnosis.

**Description, n = 4.** Adult females. **Field characters**: adult female scale nearly oval, flat, dark greyish in colour; exuviae nearly central.

**Figure 1–7.**
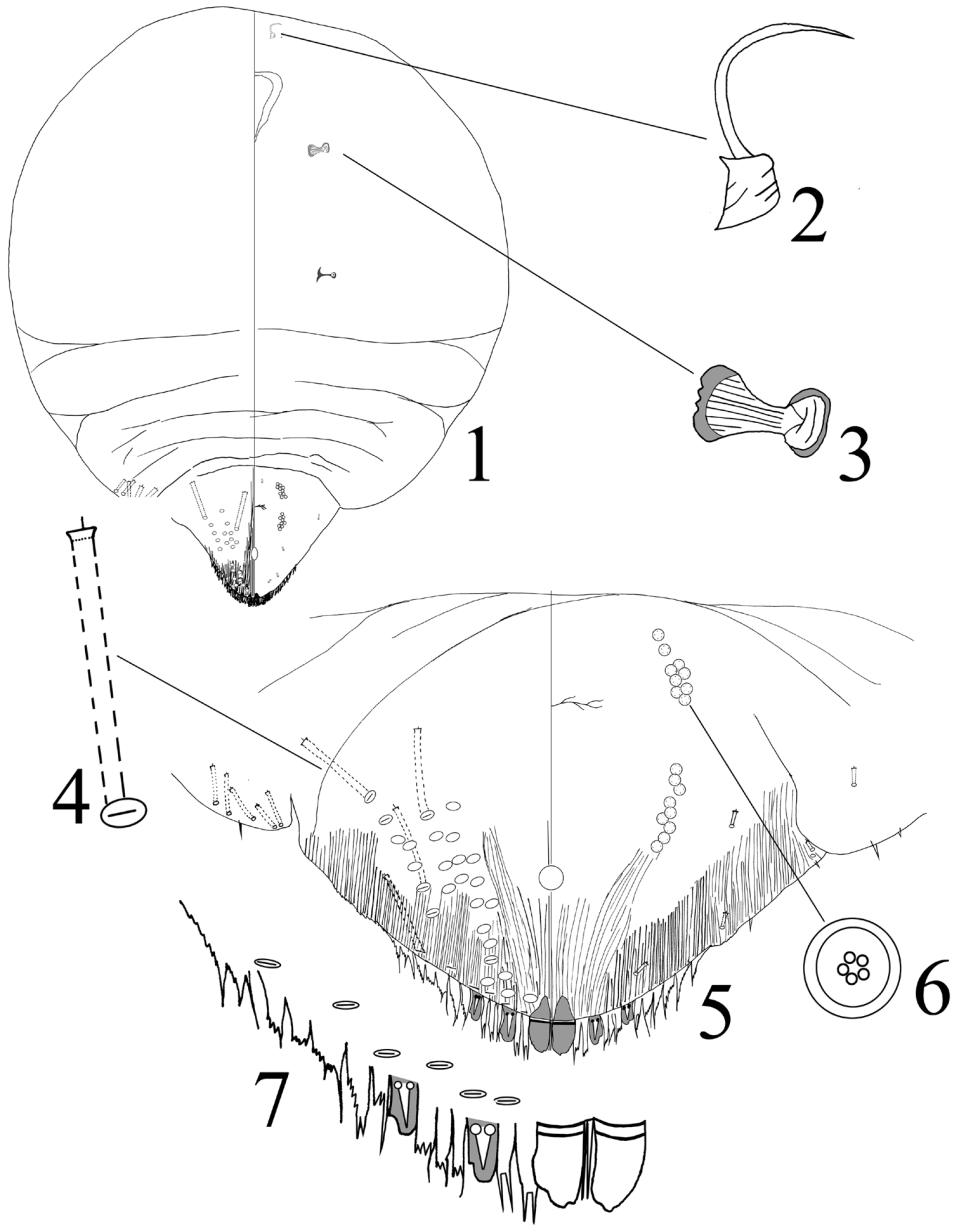
*Octaspidiotus shanghaiensis* sp. n. adult female: **1** habitus **2** detail of antenna **3** detail of anterior spiracle **4** dorsal 1-barred duct **5** pygidium **6** quinquelocular pores **7** detail of the end of pygidium margin.

**Slide-mounted**: Adult female not pupillarial, 810–952 um long (holotype 905 μm long); 756–883 μm wide (holotype is 881 μm in the widest part of the body). Body outline oval, derm membranous except for pygidium (Figure 1). **Cephalothorax.** Antennae each with 1 seta (Figure 2), distance between antennae is 164.3 μm. Prespiracular pores absent (Figure 3). **Pygidium** (Figure 5). The pygidium has three pairs of lobes: L_1_ are well-developed, a small mesal notch is present on or near the apex, and a relative larger notch is present on or near the apex of the outer margin. L_1_ is 6.7–7.2μm wide and the distance of two lobes of L_1_ is 1.5–2.1μm wide. Median lobes separated by a space 0.2–0.3 times the width of L_1_. L_2_ smaller than L_1_, with one notch on the outer margin. L_3_ similar to L_2_, but smaller. Lanceolate setae on L_2_ and L_3_ shorter than these lobes themselves. **Plates** (Figure 5 and 7) one pair of pointed plates between L_1_, not extending to the apex of the lobe; 2 pairs of plates between L_1_ and L_2_, apically fringed with few fine bifurcated; with 3 pairs of plates similar in size and shape between L_2_ and L_3_; with 6–7 pairs of plates lateral to L3. **Ducts** (Figure 4 and 5). Dorsal macroducts 1-barred-shaped. No marginal macroduct between median lobes. One marginal macroduct between L_1_ and L_2_, two between L_2_ and L_3_, and 3–4 present between L3. Dorsal submarginal macroducts about the same size as marginal macroducts which are 30–35 μm long. Total dorsal macroducts on dorsum in submarginal and marginal areas of pygidium on each side of body 32–44 (44 in holotype). Dorsal macroducts on abdomen segment IV shorter than on pygidium, with 5–6 macroducts on margin of abdomen segment IV. Ventral microducts are fewer and more scattered than the dorsal macroducts. **Anal opening** (Figure 5) 22.4–25.5 μm long in diameter, located 46.2–48.7 μm between the base of the anal opening and the base of L_1_. **Perivulvar pores** (Figure 5 and 6) present in an arc, divided in four groups, 9–12 anterolaterally and 8–9 posterolaterally.

#### Remarks.

This species is similar to *Octaspidiotus cymbidii* Tang, 1984 in the body shape and the pygidial lobes, but can be distinguished by the following characters (those for *Octaspidiotus cymbidii* in parentheses): 1) without marginal macroduct on abdomen segment III (with 3–4); 2) the three plates between L_2_ and L_3_ all equally shaped (the third plate is narrower than the first and the second plates); 3) L_1_ is separated by a space 0.2–0.3 times the width of each median lobe (by a space 0.5 times the width of each L_1_); 4) without marginal macroducts between L_1_ (present).

#### Host.

*Echinochloa crusgalli* (L.)

#### Etymology.

The specific epithet is named after Shanghai, the type locality.

#### Distribution.

China (Shanghai).

### Key to the adult females *Octaspidiotus* MacGillivray

^*^denotes Chinese species

**Table:** 

1	With 3 pairs lobes on pygidium, L_4_ absent	**2**
–	With 4 pairs lobes on pygidium, L_4_ present as small, pointed, sclerotized processes	***Octaspidiotus subrubescens* (Takahashi)**
2	Lanceolate marginal setae occurring on dorsal bases of L_2_ and L_3_ not extending to the apex of L_2_ and L_3_, respectively	**3**
–	Lanceolate marginal setae occurring on dorsal bases of L_2_ and L_3_ more-or-less extending to the apex of L_2_ and L_3_, respectively	**13**
3	All lobes hippocrepiform, without notches on margin of L_1_	*Octaspidiotus bituberculatus* Tang^*^
–	Lobes normal, with notches on margin of L_1_	**4**
4	With notches on outer margin of L_1_	***Octaspidiotus australiensis* Kuwana**
–	Notches present on both margins of L_1_	**5**
5	Three plates occurring between L_2_ and L_3_ are not equal in width	**6**
–	Three plates occurring between L_2_ and L_3_ are equal in width	**10**
6	Plates between L_1_ bifurcate or pointed apically; distance between L_1_ narrower than 1/2 of each lobe of L_1_; with 6 plates occurring lateral to L_3_	*Octaspidiotus cymbidii* Tang^*^
–	Plates between L_1_ fringed; distance between L_1_ no less than 1/2 of each lobe of L1; with no less than 7 plates occurring on the outer lateral to L_3_	**7**
7	With notches on both margins of L_3_; both second and third plates between L_2_ and L_3_ narrower than first plates between L_2_ and L_3_	*Octaspidiotus rhododendronii* (Tang)^*^
–	With notches on outer margin of L_3_, without notches on mesal margin of L_3_; Second or third plates between L_2_ and L_3_ narrower than first plates between L_2_ and L_3_	**8**
8	Second plates between L_2_ and L_3_ narrower than first and third plates between L_2_ and L_3_	**9**
–	Third plates between L_2_ and L_3_ narrower than first and second plates between L_2_ and L_3_	*Octaspidiotus yunnanensis* (Tang & Chu)^*^
9	With 22–24 perivulvar pores and 35–42 dorsal macroducts on pygidium	***Octaspidiotus tamarindi* (Green)**
–	With 43–60 perivulvar pores and 54–65 dorsal macroducts on pygidium	***O* . *tripurensis* Takagi**
10	With notches on mesal margin of L_2_; distance between L_2_ and L_3_ equal to 1/5 of each lobe of L1; plates between L_1_ bifurcate or pointed apically	*Octaspidiotus shanghaiensis* sp. n.^*^
–	With notches on both margins of L_2_; distance between L_2_ and L_3_ more than 1/3 of each lobe of L_1_; plates between L_1_ fringed	**11**
11	Body strongly sclerotized at maturity	**12**
–	Body remaining membranous	*Octaspidiotus nothopanacis* (Ferris)^*^
12	Number of perivulvar pores less than 30; with 7 plates occurring on the outer side of L_3_	*Octaspidiotus stauntoniae* (Takahashi)^*^
–	Number of perivulvar pores more than 30; with 8 plates occurring on the outer side of L_3_	***Octaspidiotus calophylli* (Green)**
13	With notches on outer margin of L_2_ and L_3_; with no more than 7 plates occurring on the outer side of L_3_	*Octaspidiotus pinicola* (Tang)^*^
–	With notches on both margin of L_2_ and L_3_; with no less than 8 plates occurring on the outer side of L_3_	**14**
14	With more than 80 dorsal macroducts and 32–47 perivulvar pores	***Octaspidiotus multipori* (Takahashi)**
–	With less than 80 dorsal macroducts and 23–29 perivulvar pores	*Octaspidiotus machili* (Takahashi)^*^

## Supplementary Material

XML Treatment for Octaspidiotus

XML Treatment for Octaspidiotus shanghaiensis
